# Supplementary material for: Dual-comb thin-disk oscillator
Source: Nat Commun. 2022 May 11;13:2584. doi: 10.1038/s41467-022-30078-0 (PMC9095605; doi:10.1038/s41467-022-30078-0)
Supplement: Supplementary file 1 — Supplementary Information [file 41467_2022_30078_MOESM1_ESM.pdf]

Supplementary Information: Dual-comb thin-disk oscillator

Kilian Fritsch[1*], Jonathan Brons[2,4], Maksim Iandulskii[2], Tobias Hofer[1], Ka Fai Mak[3], Zaijun Chen[3], Nathalie Picqué[3] and Oleg Pronin[1]

[1] Helmut-Schmidt-Universität / Universität der Bundeswehr Hamburg, Holstenhofweg 85, D-22043 Hamburg, Germany

[2]Ludwig-Maximilians-Universität München, Am Coulombwall 1, D-85748 Garching, Germany

[3] Max-Planck-Institut für Quantenoptik, Hans-Kopfermann-Str. 1, D-85748 Garching, Germany

[4] TRUMPF Laser GmbH, D-78713 Schramberg, Germany

* Corresponding author: kilian.fritsch@hsu-hh.de

Table of contents

Supplementary Tables 2
Supplementary Methods 3
Supplementary Note 7
Supplementary Discussion 8
Supplementary References 14

Supplementary Tables

Supplementary table 1: Overview of the state of the art dual-comb systems

Non-complete selection as an overview of dual-comb laser systems with high power (HP) and/or single-cavity (SC) properties.
(___) Underlined Δf_{rep} values can be easily adjusted by the user during the operation of the system.
(~) values are not directly given in the publication but were estimated from given parameters.

Publication	Ref		f_{REP} [MHZ]	Δf_{REP}	E_{PULSE}	P_{AVG}	P_{PEAK}	τ_{PULSE}	λ_0 [NM]	$\Delta\lambda$ [NM]	$\Delta\nu$ [THZ]
**This work**		SC<br>HP	60.4	up to MHz	250 nJ<br>265 nJ	15 W<br>16 W	1.2 MW<br>1.32 MW	176 fs	1030	40	11.3
**Akosman et al.**	[1]	SC	67.6	510 Hz	~89 pJ	6 mW	~0.2 kW	400 fs	1975	9.8	~0.75
**Zhao et al.**	[2]	SC	52.7	1.3 kHz	~0.3 nJ<br>~0.5 nJ	16 mW<br>25 mW	~2.7 kW<br>~2.8 kW	~105 fs<br>~160 fs	1533<br>1544	33<br>22	~4.2<br>~2.8
**Link et al.**	[3]	SC	1700	4 MHz	~35 pJ	60 mW	~2 W	18 ps	968.3	~0.1	~0.03
**Ideguchi et al.**	[4]	SC	932	325 Hz	~360 pJ<br>~330 pJ	340 mW<br>310 mW	~29 kW<br>~26 kW	12 fs	837	61	26.1
**Nürnberg et al.**	[5]	HP	2730	51.9 kHz	~8.8 pJ<br>~10.3 pJ	24 mW<br>28 mW	~21 W<br>~24 W	400 fs	1030	2.8<br>3.6	~0.8<br>~1.0
**Millot et al.**	[6]	HP	300	100 kHz	16 pJ<br>83 pJ	50 mW<br>250 mW	3.33 W<br>16.75 W	~5 ps<br>~5 ps	1530<br>1625	2<br>3	0.37
**Liao et al.**	[7]	SC	71.9	3.2 kHz	~41 pJ	3 mW	0.14 kW<br>0.16 kW	270 fs<br>250 fs	1917<br>1981	35<br>20	~2.9<br>~1.5
**Nakajima et al.**	[8]	SC	37.9	1.5 Hz	~48 pJ<br>~32 pJ	1.8 mW<br>1.2 mW	0.6 kW<br>0.4 kW	~75 fs	1550	56	7.0
**Ideguchi et al.**	[9]	HP	100	100 Hz	13 nJ	1.3 W	~600 kW	20 fs	795	~47	22
**Bernhardt et al.**	[10]	HP	130	600 Hz	~7.7 nJ	1 W (up to 17W)	~72.4 kW	100 fs	1040	30	8.3
**Mehravar et al.**	[11]	SC	72.4	82 Hz	~0.07 nJ	5.1 mW	~175 W	~370 fs	1555	9.6	1.2
**Olson et al.**	[12]	SC	60.8	229 Hz	~15 pJ	0.9 mW	~56 W	~260 fs	1865	20	1.72
**Mohler et al.**	[13]	HP	1000	2 kHz	~0.5 nJ	0.5 W	~23 kW	20 fs	795	60	28
**Sterczewski et al.**	[14]		143	Up to 10 kHz							
**Willenberg et al.**	[15]	SC	137	1 kHz	3.2 nJ	440 mW	~18.3 kW	175 fs	1050	6.5	1.77

Supplementary table 2: Comparison of thin disk and fibre technology for high-power dual-comb systems with >6 W average power

Property	Dual-comb thin-disk geometry	Dual-comb fibre geometry
**Implementation of multi-comb systems**	Yes	No
**Intensity noise**	Low high-frequency noise	Noisy at high frequencies due to multiple amplification stages
**Δf_{rep} noise**	Rather high due to free-space geometry	Rather low due to many components being monolithically integrated
**Δf_{ceo} noise**	Rather low. Our work and the work from [Neuchatel]	Rather high. See the articles cited in table 1
**Average power scalability**	Possible. Relatively low complexity. The system stays conceptually unchanged. Possible without external amplification	Possible with external amplification.
**Peak power scalability**	Possible. Relatively low complexity. The system stays conceptually unchanged. Possible without external amplification. Unfortunately, repetition rate needs to go down	Limited scalability due to high nonlinearities in fibres. Requires CPA systems, positive dispersion regime and external amplification
**Beam quality**	Usually M^2=1.1-1.2	Usually M^2=1.1-1.2
**Spectral coverage**	Mostly around 1030 nm, relatively narrowband (<20 nm). Barely available around 2000 nm	Available at 1040 nm or 1530 nm, 1620 nm, 1900 nm. Relatively broadband

Supplementary Methods

Data processing

In the following description, the dataset is a one-dimensional array containing sampling time t and signal values S. The 40 measured interferograms (bursts) are numbered by n. The 312 ms long time trace consists of 1.95×10^8 data points separated by an interval of 1.6 ns. This corresponds to a sampling rate of 625 MSa/s. The trace is recorded with 14-bit vertical resolution (oscilloscope Keysight DSOS254A).

Burst arrival time correction

The optical spectrum was reconstructed from the temporal signal (the set of interferograms) with the help of the discrete Fourier transformation DFT (pyFFTW). DFT was applied to the whole dataset. However, additional numerical data processing (phase correction) is necessary to compensate for the fluctuations of the Δf_{rep} or in other words, a timing jitter between the interferograms. This jitter was corrected numerically with the following method. (Supplementary Fig. 1 shows burst arrival time correction results for 40 bursts.)

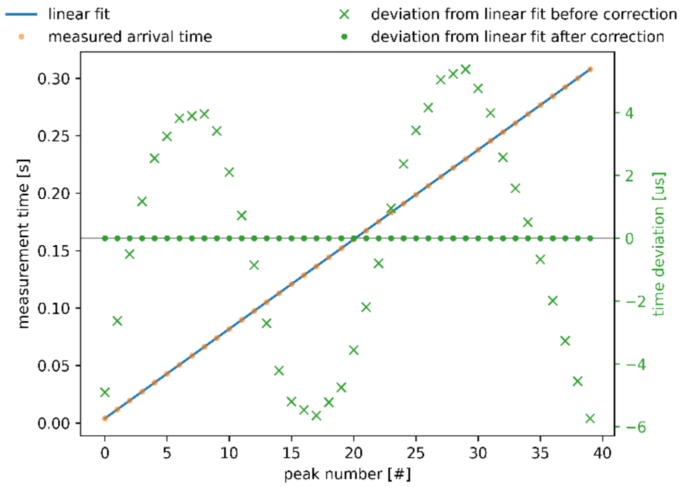

Supplementary Fig. 1 Burst arrival time correction. The burst arrival time is displayed on the left axis, the difference between linear fit and actual arrival time is shown on the right axis.

Step 1: Assessment of the deviation. By finding the array index of the maximum of each burst $\mathrm{idx}_{\mathrm{peak}}(n)$ (with SciPy's peak finding algorithm scipy.signal.find_peaks) in the time signal, the interferograms' arrival time can be measured with $\tau_{\mathrm{measured}}(n) = t(\mathrm{idx}_{\mathrm{peak}}(n))$. This value could be fitted linearly with the function t_{fit}. The slope

should be the inverse of the difference in repetition rate. Evaluating the fit function at the burst index yields the expected interferogram arrival time $\tau_{\text{fit}}(n) = t_{\text{fit}}(\text{idx}_{\text{peak}}(n))$.

Step 2: Calculating a correction factor. The ratio of expected arrival time (taken from the linear fit) to the measured arrival time serves as a multiplicative correction factor:

$$\zeta'^{(n)} = \frac{\tau_{fit}(n)}{\tau_{measured}(n)}$$

However, this correction factor is only well defined at the maximum of each burst, because in between bursts no arrival time can be measured. Nevertheless, it must be applied to the entire time vector of the measurement, hence it is to be interpolated to the continuous correction factor ζ. In this case, a simple univariate spline (scipy.interpolate.UnivariateSpline) of cubic order ($k = 3$) was chosen (see Supplementary Fig. 2). No spline smoothing was applied ($s = 0$), i. e., all data points are knots of the spline.

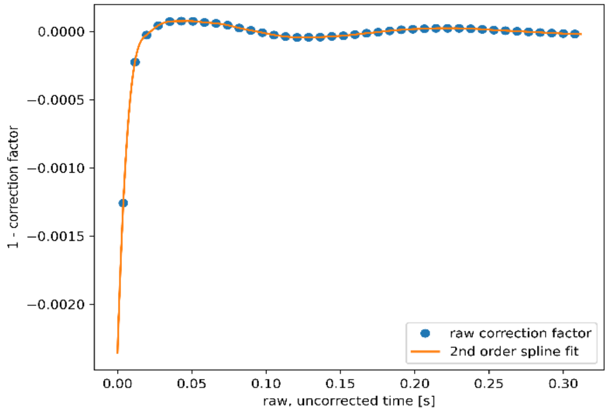

Supplementary Fig. 2 Arrival time correction factor and spline fit. The blue dots show the correction factor for beach interferogram, the orange curve provides the 2nd order continuous spline fit.

Step 3: Applying the correction. The time vector may now be corrected by multiplying it by the correction factor: $t_{\text{corrected}} = \zeta \cdot t$. However, the corrected time vector does not correspond to the signal values anymore. This must be accounted for by interpolating the signal values onto the new temporal vector with SciPy's interpolate function (scipy.interpolate.interp1d). Here, the linear interpolation algorithm was used. Note that the corrected time vector is not evenly spaced anymore. To find the frequency vector after the DFT, the temporal spacing of the uncorrected time vector must be used. This is correct, since it corresponds to the sampling rate of the DAQ system.

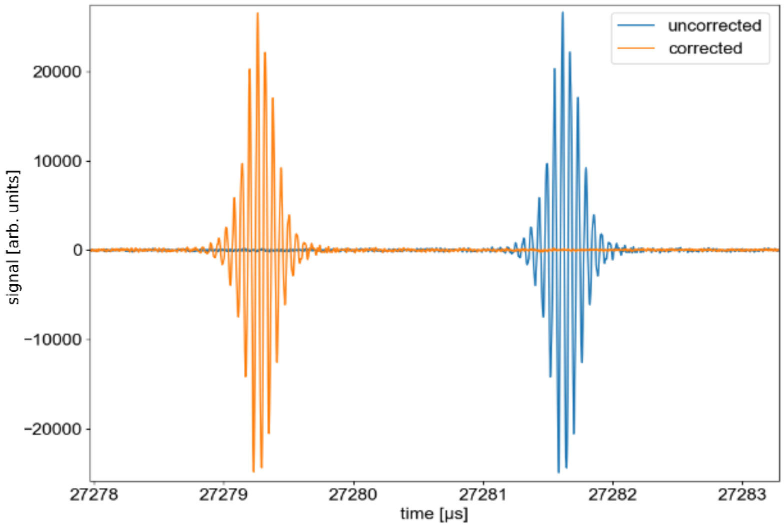

Supplementary Fig. 3 Time corrected and interpolated Interferogram. After applying the correction method, the blue interferogram was shifted in time to the new position (orange interferogram,).

Evaluation of the time correction. To assess the performance of this correction algorithm, one may compare the deviation between the linear fit and the actual burst arrival time before and after the correction in Supplementary Fig 1. Initially, the absolute deviations between expected and measured burst are 11.1 µs peak to peak. After the correction algorithm was applied, these deviations are suppressed by a factor of 4270 down to 2.6 ns peak to peak. Note that the data were sampled in 1.6 ns intervals, so the remaining fluctuations correspond to two sampling intervals. The 11.1 µs peak-to-peak fluctuation corresponds to a relative fluctuation of 1.4×10^{-3}, taking into account the 7.8 ms separation between interferograms. This, in turn, corresponds to approximately 0.18 Hz peak to peak or 59 mHz standard deviation when the Δf_{rep} is tuned to 128 Hz.

Apodization

Significant satellite interferograms corresponding to side-pulses in the mode-locked trace can be observed, see Supplementary. Fig 11. In addition, there is significant noise between bursts. It has a purely electronic origin and may be filtered out. Apodization is a well-known technique used in such situations. For apodization, a super-gaussian local filter function of order o and width w at position c_n with amplitude one was used:

$$f_\mathrm{n}(t) = \exp\left\{\frac{-2^{2o}\ln(2)\cdot(t-c_n)^{2o}}{w^{2o}}\right\}$$

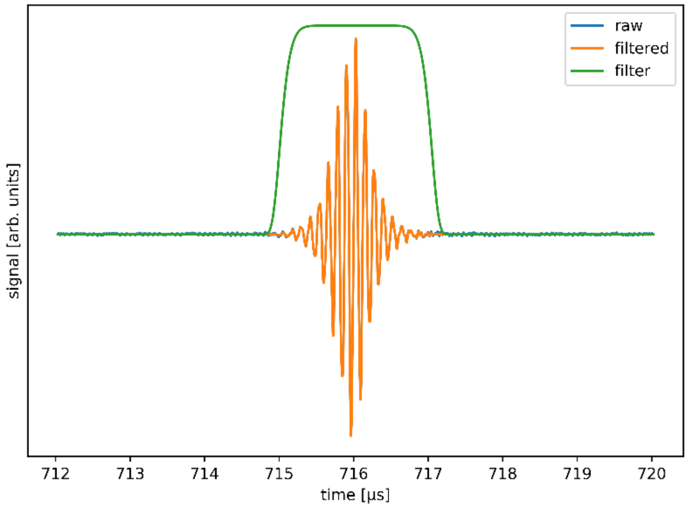

Supplementary Fig. 4 Interferogram apodization. Example of the apodization process showing the original data (blue), filter data (orange), and the local filter function (green). Note that the filter amplitude was scaled up to 6000 to be visible in the graph.

In the presented measurements, the filter was parameterized with $o = 6, w = 2\ \mu s$. The location of each burst $c_\mathrm{n}$was found numerically by SciPy's peak finding algorithm. If more than one interferogram is in the data set, the sum of all local filter functions is used:

$$F_\mathrm{total}(t) = \sum\nolimits_\mathrm{n} f_\mathrm{n}(t)$$

The corrected signal is simply $S_\mathrm{apodized} = F_\mathrm{total}(t)\cdot S_\mathrm{raw}$. The multiplication here is carried out element-wise.

Supplementary Note

Acetylene spectroscopy measurement setup

We have chosen acetylene gas for a "real-life" test of our thin-disk dual-comb spectrometer. The laser spectrum was shaped with the help of a bandpass filter (central wavelength 1030 nm, FWHM 10 nm) and a slightly tilted long-pass filter (Thorlabs FELH1050). In our setup, the tilt was set to spectrally select the laser output around 1034nm. Because significant power is lost in this filtering process, a flexible power attenuation setup with half waveplates and thin-film polarizers is implemented in the beam paths. The power is adjusted to maximize the signal on the photodiode without oversaturating it. The spectrally filtered laser radiation enters the 52 cm-long acetylene cell pressurized at 1.4 bar, and then it is sampled by a photodiode.

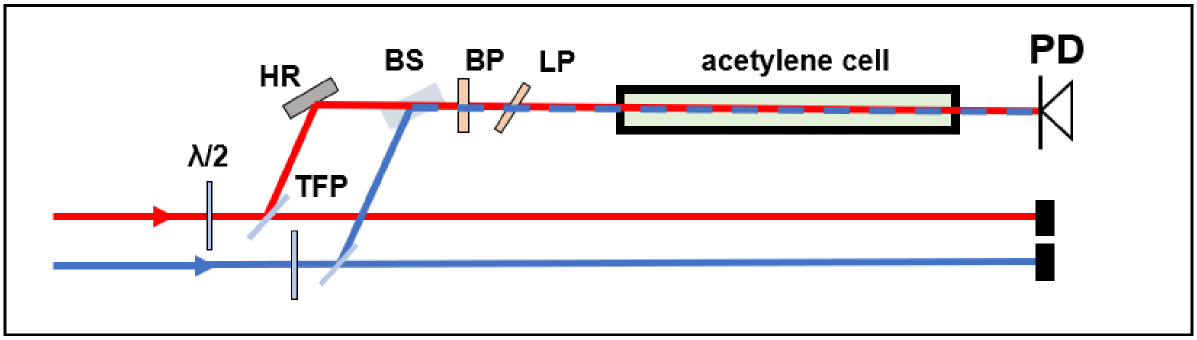

Supplementary Fig. 5 Acetylene spectroscopy setup. The two frequency combs (red, blue) enter the spectroscopy setup from the oscillators on the left side. For flexible power scaling through the interferometer, half-waveplates (λ/2) and thin-film polarizers are placed into the path of the beams. Both beams are overlapped with a high reflective mirror (HR) and a beam splitter (BS). The spectrum is narrowed with a band pass filter (BP), and the central laser wavelength is shifted with a long pass filter (LP) towards the absorption wavelengths of acetylene. Then the beam enters the 52cm acetylene cell at 1.4 bars of pressure, before it is sampled with a photo diode (PD).

Supplementary Discussion

Performance of the data correction

This section summarizes how the data evaluation is influenced by both correction methods, arrival time correction and apodization. All temporal signals are zero-padded to double their length to interpolate the spectrum. First, Supplementary Fig. 6 depicts a measurement containing 40 interferograms at 128 Hz Δf_{rep} without any corrections

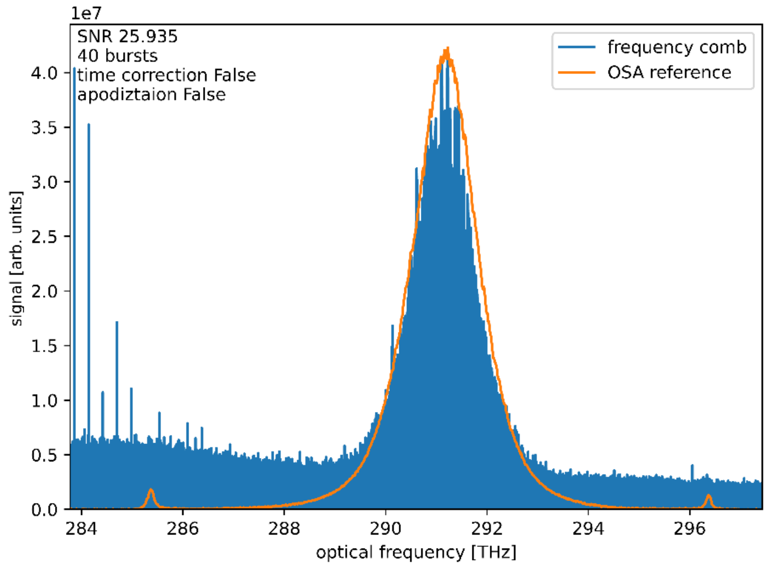

Supplementary Fig. 6 Uncorrected spectrum. The orange curve shows the OSA reference. The FFT which was frequency shifted to the optical domain is displayed in blue.

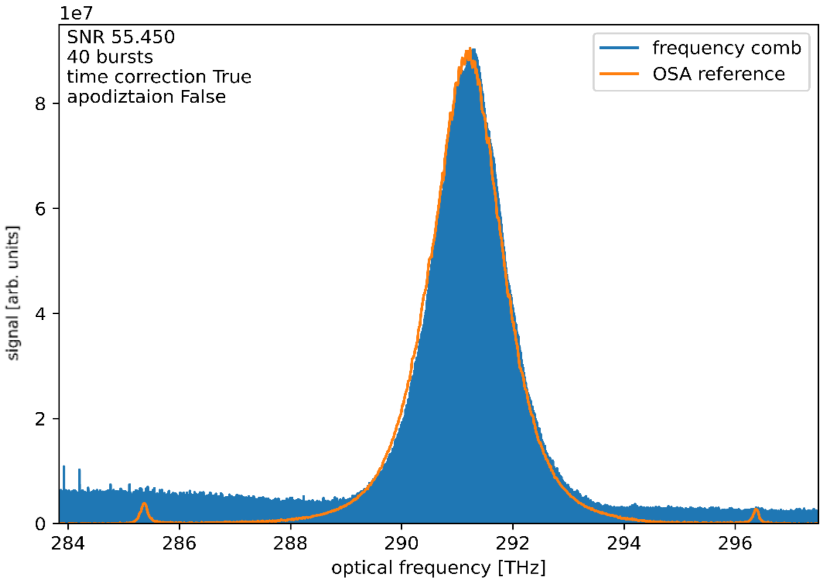

Supplementary Fig. 7 Spectrum of time corrected data. The orange curve shows the OSA reference. The FFT of the time corrected time trace is shown in blue.

applied. Note that the signal-to-noise ratio (SNR) of 25.9 is relatively low, and the spectral shape is substantially buried in the noise floor. SNR is defined as a ratio of the spectral peak magnitude to the background signal mean value. The mean value of the background signal is calculated in 1-3.5 MHz range in RF domain or 238.8-285 THz in optical domain. In Supplementary Fig. 7, the arrival time correction algorithm was applied but without the

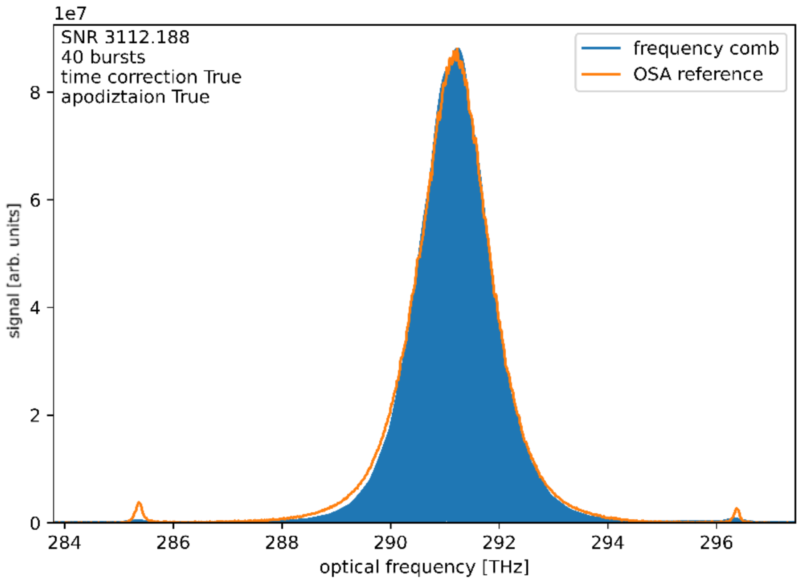

Supplementary Fig. 8 FFT of time corrected and apodized data. The orange curve shows the OSA reference, the FFT of the time corrected and apodized time trace is shown in blue.

apodization. A two-fold improvement in $\mathrm{SNR}_{\mathrm{time\ corr.}}$ to 55 and a clear agreement of the reference spectrum and the dual-comb spectrum can be observed.

Finally, applying both apodization and arrival time correction yields Supplementary Fig. 8. Here, the background noise is practically eliminated from the measurement, and the $\mathrm{SNR}_{\mathrm{time\ corr.-apod.}}$ is improved by a factor of almost 56 to 3112.

The spectral shapes of reference measurement and dual-comb measurement clearly agree. Even the weak spectral Kelly sidebands at around 285.5 and 296.5 THz, initially buried in the noise, appear after applying the time correction and apodization. Supplementary Fig. 9 displays SNR versus the number of bursts for timing jitter-corrected and non-corrected measurements. Signals have been apodized in both cases. SNR scales with $\sqrt{n}$ bursts, which is expected for uncorrelated noise.

Central frequency drift.

A drift in the center frequency of a spectrum corresponding to an individual interferogram is directly related to a drift in the difference in the carrier-envelope-offset frequencies between the two frequency combs. To assess this quantity, the DFTs of 4 sets of 10 individual bursts were analyzed. The sets are disjointed, i.e. the first set contains the interferograms 1 through 10, the second contains the interferograms 11 through 20, and so on. The raw data containing eleven interferograms is 80 μs long. To distinguish the location of the comb lines, the data were symmetrically zero-padded to a length of 0.93 s. A total of 40 interferograms were considered in the evaluation.

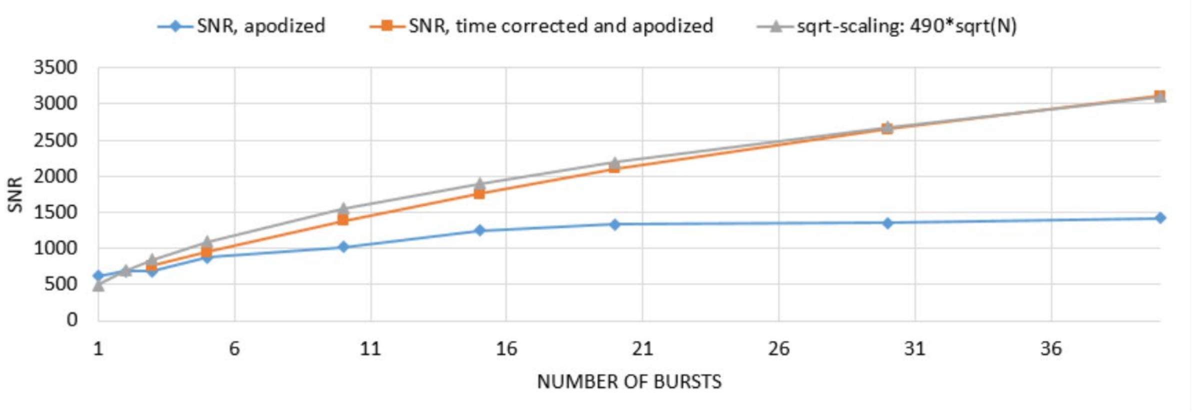

Supplementary Fig. 9 SNR vs measurement time (number of bursts). SNR scales as the square root of the number of bursts as shown with the orange line (time corrected and apodized data) The not time-corrected SNR value is displayed in blue.

Arrival time correction and apodization were applied. Supplementary Fig. 10 a) shows the raw data of the first set of 10 bursts. Supplementary Fig. 10 b) shows the DFT spectra in the RF domain. Supplementary Fig. 10 c) is a zoomed-in view of the comb line centered at 16.686 MHz and its neighbors. In b) and c), the orange markers highlight the location of the comb line's peak. Supplementary Fig. 10 d) Shows the drift in center frequency over all

4 sets of bursts. The $\mathrm{SNR}_{\mathrm{time\ corr.-apod.}}$ for 10 apodized and time-corrected bursts is approximately 1380. The vertical red line represents the mean value at 16.7 MHz and the grey box around it is as wide as one standard deviation of the dataset, 0.38 Hz. Taking into account the well-known equation and $\Delta f_{CEO} = 0.38\ Hz$:

$$\Delta f_{\mathrm{CEO}} = \frac{\Delta\varphi_0 \Delta f_{\mathrm{rep}}}{2\pi}$$

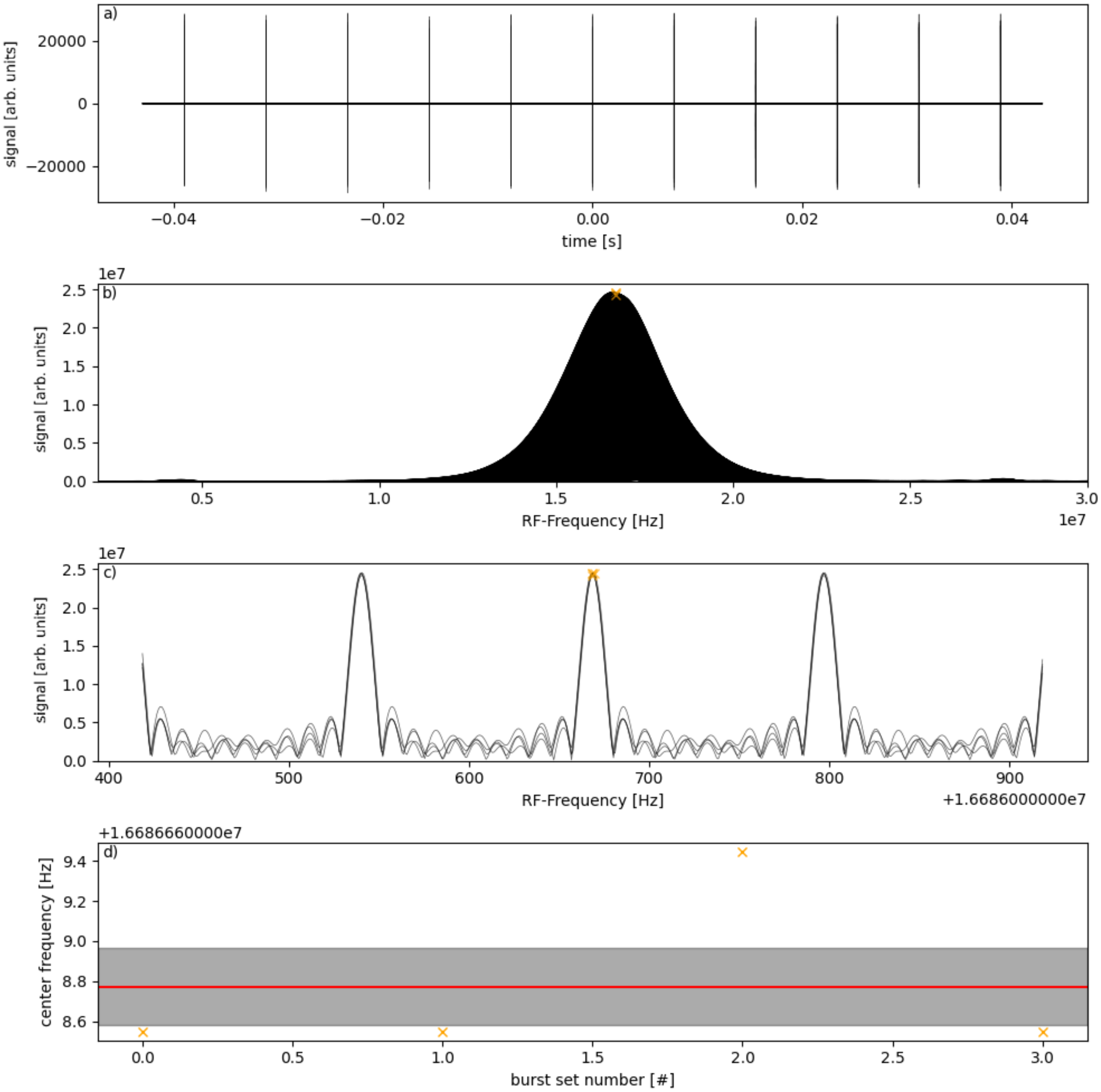

Supplementary Fig. 10 Drift of the center frequency of the spectrum of 4 sets of 10 interferograms. a) time trace of the frist burst set. b) DFT in the RF domain of all sets. c) Zoom in view of b) ob the center comb line and its neighbours. d) center frequency of the center comb line of each RF spectrum. The vertical red line represents the mean value of all center frequencies at 16.7MHz, the grey box around has the width of one standard deviation of 0.38 Hz. Burst arrival time correction and apodization is applied in this analysis. Before the DFT the signal is zero-padded to a length of 1.2 s from initially 0.1 s containing the ten interferograms.

we can calculate the relative CEP noise $\Delta\varphi_0$ to be12 mrad.

Tuning the repetition rate difference

Initially, we were interested in the detuning range 100 Hz–1 kHz particularly relevant for the dual-comb spectroscopy. For very narrow spectra, higher Δf_{rep} values could be of interest, so we experimentally verified that our dual-comb laser source can easily be tuned within this range and can be detuned even up to Δf_{rep}= 1 MHz at $f_{\mathrm{rep}} = 60$ MHz. Practically there is no limit for the lower detuning value. However, there can be a limit for the upper detuning value. There are two possible reasons for it. 1. The detuning of one of the cavities changes the roundtrip time and, thus the pulse train repetition rate. This, in turn, influences the pulse energy $E_{\mathrm{p}} = f_{\mathrm{rep}} \cdot P_{\mathrm{avg}}$. Since all optical elements are shared in the laser cavities (Kerr medium, separation between the focusing curved mirrors), Kerr-lens mode-locking is optimized for only a certain pulse energies range. Considering our previous experiments, we can estimate that a 5 % energy decrease (or increase) will lead to instabilities in one of the arms. This 5 % change in the cavity length corresponds to approximately 3 MHz maximal acceptable detuning. 2. The detuning of one of the cavity arms changes the stability zone of this cavity. As Kerr-lens mode-locking is quite sensitive to the cavity position in the stability zone, this will manifest itself in a decreased pulse energy of the detuned cavity up to the point where this cavity will cease mode-locking. These limits depend strongly on the specific cavity configuration.

Analysis of temporal side pulses

Satellite pulses are a well-known phenomenon in mode-locked lasers. In DCS they can lead to satellite interferograms which might disturb spectroscopic measurements. In our thin disk oscillator satellite pulses were present and measured with a a autocorrelator (APE pulseCheck). In both, the top and bottom cavity, satellite pulses appear with around 4 picoseconds temporal distance to the main pulse which is displayed in Supplementary Fig. 11a). The main pulse measured to be of 178fs duration (FHWM, sech² fit) for our top cavity – the bottom cavity shows similar behaviour. These pulses lead to satellite interferograms as displayed in Fig. 11b), taken from a measurement set with Δf_{rep}= 188 Hz and Δf_{rep}= 61.1 MHz.

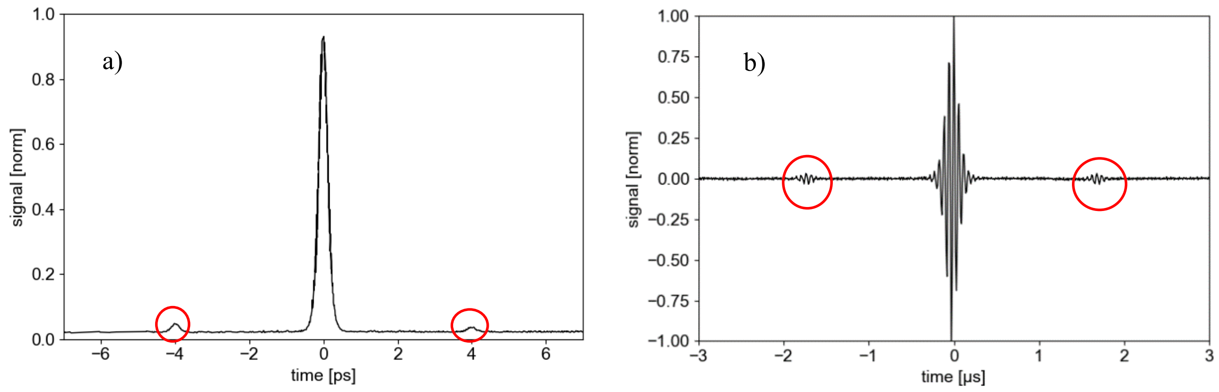

Supplementary Fig. 11 Satellite pulses and satellite interferograms. a) the two satellite pulses can be seen in an autocorrelator-measurement of our top-cavity with 4 ps timing difference to the main pulse. b) The resulting satellite interferograms.

The temporal position τ_{sat} of in the downconverted satellite interferogram with respect to the main interferogram can be estimated with the down-conversion factor *a* and the real side-pulse difference in timing 4 ps:

$$\tau_{\mathrm{sat}} = \mp \frac{\mathrm{f_{rep}} * 4\,ps}{\Delta f_{\mathrm{rep}}} = \mp \frac{4ps}{a}$$

Fortunately, with careful adjustment of oscillator parameters such as mirror dispersion and pump power, it was possible to suppress the side-pulses for the presented measurement set. In addition to the two satellite interferograms shown in Supplementary Fig. 11b), the side pulses beat with themselves and create weak satellite interferograms 3 and 4 which in our case were probably burried in noise.

Supplementary References

1. A. E. Akosman, and M. Y. Sander, "Dual comb generation from a mode-locked fiber laser with orthogonally polarized interlaced pulses," Optics express **25**, 18592–18602 (2017).
2. X. Zhao, G. Hu, B. Zhao, C. Li, Y. Pan, Y. Liu, T. Yasui, and Z. Zheng, "Picometer-resolution dual-comb spectroscopy with a free-running fiber laser," Optics express **24**, 21833–21845 (2016).
3. S. M. Link, D. J. H. C. Maas, D. Waldburger, and U. Keller, "Dual-comb spectroscopy of water vapor with a free-running semiconductor disk laser," Science (New York, N.Y.) **356**, 1164–1168 (2017).
4. T. Ideguchi, T. Nakamura, Y. Kobayashi, and K. Goda, "Kerr-lens mode-locked bidirectional dual-comb ring laser for broadband dual-comb spectroscopy," Optica **3**, 748 (2016).
5. J. Nürnberg, C. G. E. Alfieri, Z. Chen, D. Waldburger, N. Picqué, and U. Keller, "An unstabilized femtosecond semiconductor laser for dual-comb spectroscopy of acetylene," Optics express **27**, 3190–3199 (2019).
6. G. Millot, S. Pitois, M. Yan, T. Hovhannisyan, A. Bendahmane, T. W. Hänsch, and N. Picqué, "[Duplikat] Frequency-agile dual-comb spectroscopy," Nature Photon **10**, 27 EP - (2016).
7. R. Liao, Y. Song, W. Liu, H. Shi, L. Chai, and M. Hu, "Dual-comb spectroscopy with a single free-running thulium-doped fiber laser," Optics express **26**, 11046–11054 (2018).
8. Y. Nakajima, Y. Hata, and K. Minoshima, "High-coherence ultra-broadband bidirectional dual-comb fiber laser," Optics express **27**, 5931–5944 (2019).
9. T. Ideguchi, S. Holzner, B. Bernhardt, G. Guelachvili, N. Picqué, and T. W. Hänsch, "Coherent Raman spectro-imaging with laser frequency combs," Nature **502**, 355 EP - (2013).
10. B. Bernhardt, A. Ozawa, P. Jacquet, M. Jacquey, Y. Kobayashi, T. Udem, R. Holzwarth, G. Guelachvili, T. W. Hänsch, and N. Picqué, "Cavity-enhanced dual-comb spectroscopy," Nature Photon **4**, 55 EP - (2010).
11. S. Mehravar, R. A. Norwood, N. Peyghambarian, and K. Kieu, "Real-time dual-comb spectroscopy with a free-running bidirectionally mode-locked fiber laser," Appl. Phys. Lett. **108**, 231104 (2016).
12. J. Olson, Y. H. Ou, A. Azarm, and K. Kieu, "Bi-Directional Mode-Locked Thulium Fiber Laser as a Single-Cavity Dual-Comb Source," IEEE Photon. Technol. Lett. **30**, 1772–1775 (2018).
13. K. J. Mohler, B. J. Bohn, M. Yan, G. Mélen, T. W. Hänsch, and N. Picqué, "Dual-comb coherent Raman spectroscopy with lasers of 1-GHz pulse repetition frequency," Optics letters **42**, 318–321 (2017).
14. Ł. A. Sterczewski, A. Przewłoka, W. Kaszub, and J. Sotor, "Computational Doppler-limited dual-comb spectroscopy with a free-running all-fiber laser," APL Photonics **4**, 116102 (2019).

15. B. Willenberg, J. Pupeikis, L. M. Krüger, F. Koch, C. R. Phillips, and U. Keller, "Femtosecond dual-comb Yb:CaF2 laser from a single free-running polarization-multiplexed cavity for optical sampling applications," Optics express **28**, 30275–30288 (2020).
